# Spatial analysis of cluster randomised trials: a systematic review of analysis methods

**DOI:** 10.1186/s12982-017-0066-2

**Published:** 2017-09-21

**Authors:** Christopher Jarvis, Gian Luca Di Tanna, Daniel Lewis, Neal Alexander, W. John Edmunds

**Affiliations:** 1 London School of Hygiene and Tropical Medicine, London, UK; 2 MRC London Hub for Trials Methodology Research, London, UK; 3 Queen Mary University of London, London, UK

**Keywords:** Cluster randomised trials, Spatial effects, Spatial analysis, Spillover, Systematic review

## Abstract

**Background:**

Cluster randomised trials (CRTs) often use geographical areas as the unit of randomisation, however explicit consideration of the location and spatial distribution of observations is rare. In many trials, the location of participants will have little importance, however in some, especially against infectious diseases, spillover effects due to participants being located close together may affect trial results. This review aims to identify spatial analysis methods used in CRTs and improve understanding of the impact of spatial effects on trial results.

**Methods:**

A systematic review of CRTs containing spatial methods, defined as a method that accounts for the structure, location, or relative distances between observations. We searched three sources: Ovid/Medline, Pubmed, and Web of Science databases. Spatial methods were categorised and details of the impact of spatial effects on trial results recorded.

**Results:**

We identified ten papers which met the inclusion criteria, comprising thirteen trials. We found that existing approaches fell into two categories; spatial variables and spatial modelling. The spatial variable approach was most common and involved standard statistical analysis of distance measurements. Spatial modelling is a more sophisticated approach which incorporates the spatial structure of the data within a random effects model. Studies tended to demonstrate the importance of accounting for location and distribution of observations in estimating unbiased effects.

**Conclusions:**

There have been a few attempts to control and estimate spatial effects within the context of human CRTs, but our overall understanding is limited. Although spatial effects may bias trial results, their consideration was usually a supplementary, rather than primary analysis. Further work is required to evaluate and develop the spatial methodologies relevant to a range of CRTs.

**Electronic supplementary material:**

The online version of this article (doi:10.1186/s12982-017-0066-2) contains supplementary material, which is available to authorized users.

## Background

Randomised controlled trials assess the efficacy and safety of interventions [1, 2]. When it is difficult to allocate interventions at the individual level, for example due to logistical or financial restrictions, randomisation and allocation of interventions at a group level may be preferred, this is a cluster randomised trial (CRT) [3]. CRTs also allow for estimation of spillover and herd effects; the apparent treatment effect on individuals who do not receive the intervention [4]. Failure to account for spillover effects can result in biases that reduce the quality of trials and mean that absence of bias is no longer guaranteed by the randomisation, especially when the relationship between intervention and outcome is complex [5, 6].

Spatial effects are effects stemming from locational variation in the distribution of phenomena of interest or in the intensity of interaction between phenomena of interest. Such effects manifest as local variation in the estimated treatment effect over a study area. The existence of spatial effects suggests that global effects estimates are uncertain, and may under- or over-estimate the true effect dependent on location. When values of a single variable are related to nearby values of the same variable this is called spatial dependence, the existence of which is usually captured using spatial autocorrelation measures [7]. Spatial dependence is a fundamental concept in spatial statistics [8] and stems from Tobler’s [9] 1st law of geography that “everything is related to everything else, but near things are more related than distant things.” In agricultural field trials it is long established that the location of the data can impact on trial results [10]. Incorporation of spatial methodology in agricultural trials is common, [11] but the impact of spatial effects in human CRTs have not been researched extensively.

Clusters in CRTs are often defined geographically and valid inference relies on the assumption that the clusters are independent irrespective of their nearness to one another [3]. There is frequently an assumption of an absence of spillover; that movement of people and diseases occurs freely within a cluster but movement between clusters is negligible, non-existent, or not relevant [12]. This assumption can be violated when there is movement of people or diseases across borders, such as mosquitoes flying between control and intervention households. If an intervention such as insecticide-treated bed nets provide a protective effect to nearby control households then ignoring mosquito mobility will result in underestimating the intervention effect of the trial. This could result in trials discarding effective interventions because the control and intervention are both receiving the benefit of the treatment.

Spillover may also be due to connections in social networks, [13] also violating the assumption of independence. In this paper, we consider spillover that can be estimated using GPS data which is often collected as part of trials and therefore do not consider social networks. Spillovers are more likely in trials that have spatially close clusters, and the effect is especially important when an individual’s outcome is affected by their proximity to other individuals with different exposure statuses.

One way to minimise the potential for spillover is to design a trial with well-separated clusters. In practice this may not be logistically or financially feasible as spatial effects can be present over distances of several kilometres [14]. Furthermore, greater distances between clusters removes our ability to measure spillover spatial effects. To be able to measure spatial effects we need to have data on people nearby to one another and by separating the clusters we may no longer have such information. If proximity to an intervention affects non-treated individuals this is of usually scientific interest and something we should measure. There is a need to control for and estimate spatial effects in the analysis of a trial without adding extra complexity to the design.

We therefore focus only on spatial analysis methods used in CRTs in this review and do not consider alternative trial designs. A further reason for focusing on analysis methods is that this may enable analysis of existing and previous CRTs where redesign is not possible. This review is a diagnostic review of spatial analysis methods that have been used in CRTs. As such, it does not attempt to pose statistical solutions or determine the best way to account for spatial effects within CRTs. We will describe the state of the literature and aim to improve understanding and help inform further research into spatial effects within CRTs by (1) Identifying spatial analysis methods used in CRTs. (2) Summarising and grouping spatial methods (3) Assessing the impact of spatial effects.

## Methods

### Search terms and review process

The PRISMA guidelines [15] for systematic reviews and meta-analyses were followed for this review. It was conducted between January 2016 and September 2016. Ovid/Medline, Pubmed, and Web of Science databases were electronically searched and Mendeley was used to store articles. Search terms for CRTs and spatial effects are detailed in Table 1. Studies up to end of 2015 were included and only English language articles and search terms were considered.

**Table 1:** Search term

Databases	CRT terms	Spatial terms	Search string
Pubmed	Randomi*ed Trial	Spatial*	(Randomi*ed trial) AND (group OR community OR cluster OR place) AND (spatial* OR indirect effect* OR spillover* OR contamination* OR externalit*)
Medline/Ovid	Group	Indirect effect*	
WebOfScience	Community	Spillover*	
	Cluster	Contamination*	
	Place	Externalit*	

A star (*) represents a wildcard character

Papers from each database were combined into a single spreadsheet containing the title, authors, journal, and year. Duplicates were removed automatically within the software and then manually during the title screen. The titles were screened to remove irrelevant papers such as individually randomised trials. Following this, abstracts were screened and the full texts of potentially relevant papers were independently reviewed by two reviewers and disagreements resolved. After selecting relevant papers the references of the articles were screened.

### Inclusion and exclusion criteria

The inclusion criteria for studies were: (1) the study is a cluster randomised trial. (2) Spatial methods are used in the analysis of the study. We categorised a spatial method as one which accounts for the structure, location, or relative distances of the data. This includes direct estimation of an effect such as, the change in risk for those within 100 m of an intervention household or the use of a spatial model which account for spatial structure.

The exclusion criteria were (1) non-randomised studies (2) individual randomised trials and hybrid CRTs such as Double or pseudo-randomised studies as they were considered not to be cluster randomised trials (3) grey literature (4) studies where spillover effects are measured in a non-spatial way, for example comparison of vaccinated and non-vaccinated individuals within an intervention cluster. (5) Studies that account for spatial effects at the design stage only; for instance, using buffer zones or well-separated clusters (6) articles which were study protocols and therefore had not applied their methods yet.

### Data extraction

The following variables were collected on each paper: title, year, journal, author, intervention, outcome, whether a map was presented, spatial analysis method.

## Results

### Search results

**Fig. 1:**
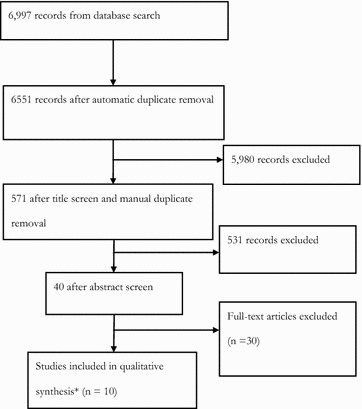
Flow chart of search results

A flow chart of the search process can be seen in Fig. 1 and the search terms in Table 1. The search terms returned 6997 records, reducing to 571 records after the title screen and duplicate removal. Of the 571 records, 40 abstracts were considered relevant for full text review by the reviewers. One study [16] was a replication analysis and it was decided to include the original study in the review instead of the replication. There are ten papers and thirteen trials in this review as some papers include multiple trials.

Whilst this review was being conducted a systematic review on health-related spillover in impact evaluations was released [13]. It has the more general aim of attempting to summarise methods to estimate health related spillover in low and middle income countries. Our review is different as it only includes spatial methods used within cluster randomised trials and does not restrict by type of country. The results from both reviews were compared and did not lead to additional records being included.

### General characteristics

This review contains ten papers published between 1998 and 2015, they relate to thirteen trials as some papers contained more than one trial. The trials took place around the globe with three taking place in Kenya, three in the United Kingdom, and the rest in Mexico, Venezuela, Ghana, Papua New Guinea, Vietnam, Haiti, and India. There is one stepped wedged trial [17] and all others are parallel cluster randomised trials. Six of the papers were a spatial reanalysis of a previously reported trial.

Seven trials focused on pathogens carried by mosquitoes. The intervention for six of these was insecticide treated bed nets or curtains and the other intervention was a drug. Two of the seven mosquito trials considered all cause child mortality as an endpoint, the other five looked at entomological endpoints.

There were two vaccine trials, the first looked at vaccine uptake in response to a mass campaign and the second evaluated vaccine effectiveness for a typhoid vaccine. One paper considered primary care and community based trials, within this they applied spatial methods to three different trials, one simulated and two real trials. The final trial looked at the impact of deworming on education and health within schools. Further details of the trails in the review are in Table 2 and in Additional file 1: Table S1.

**Table 2:** Characteristics of the included papers

Author and year	Location	Intervention	Outcome	Map	Spatial method type	Spatial method
Binka 1998 [22]	Ghana	Permethrin-impregnated bed net	All-cause child mortality	Yes	Straight line distance	Distance to discordant observation and points of interest. Standardised mortality rates calculated at several distances
Alexander 2003 [27]	Papua New Guinea	Diethylcarbamazine (DEC) plus ivermectin versus DEC alone	Spatial distribution of Wuchereria bancrofti and microfilaraie	Yes	Spatially structured random effect	Negative binomial model with a distance parameter in the covariance structure of a random effect. Measures half distance of spatial correlation
Gimnig 2003 [19]	Kenya	Permethrin-treated bed net	Spatial distribution of mosquitoes	Yes	Straight line distance	Distance to discordant observation and to points of interest. Poisson regression model with a random effect for cluster
Hawley 2003 [18]	Kenya	Permethrin-treated bed net	All-cause child mortality, anaemia, and density of mosquitoes	Yes	Straight line distance	Distance to discordant observation and to points of interest. Cox regression model with a random effect for adjusted for cluster
Miguel and Kremer 2004 [23]	Kenya	Deworming	Helminth infection	No	Density	Total number and proportion of treated students within 6 km of a school. Included in primary analysis random effects model adjusted for cluster (School)
Kroeger 2006 [20]	(1) Mexico (2) Venezuela	Insecticide-treated curtains and water container covers	Reduction in entomological indices for Dengue	No	Straight line distance	Distance to nearest participant with outcome at the beginning of the study Odds ratio of outcome for nearby houses compared to houses further away
Ali 2007 [21]	Vietnam	Vaccine campaign	Vaccine uptake	Yes	Straight line distance and density	Distance to points of interest and density. Random effects mode including typhoid prevalence and private practitioner density
Lenhart 2008 [24]	Haiti	Insecticide-treated bed nets	Reduction in entomological indices for Dengue	Yes	Density	Total number of bed net households within 100 m. Spearman’s correlation of number of bed net houses within 100 m compared with change in entomological measures
Silcocks 2010 [29]	UK (3 trials)	(1) Sun exposure, (2) home safety intervention, (3) intervention to reduce baby walker use	(1) Lip cancer, (2) number of injuries per individual, (3) ownership of baby walker	No	Spatially structured random effect	Spatial weights matrix in covariance of random effect. A multiple membership spatial random effects model with fixed North/South or East/West gradient covariate effect for gradient
Chao 2015 [25]	India	Typhoid vaccine	Vaccine effectiveness	Yes	Density	The sum of the risk of those within 100 m of a participant called the potential exposure. Included in a model with a random effect for cluster

Five trials used a two stage method in their primary analysis, the rest a one stage method [3]. Seven papers included a map of the study location. The software used included Fortran, SAS, ArcGIS, HLM, Stata, MLWin, R, and WinBugs, four studies did not report software used. There were two methodology papers (four trials) that focused on how to adjust for spatial effects, and eight applied papers (nine trials) where analysis of the spatial effect was not the primary aim of their trial.

### Spatial methods

**Fig. 2:**
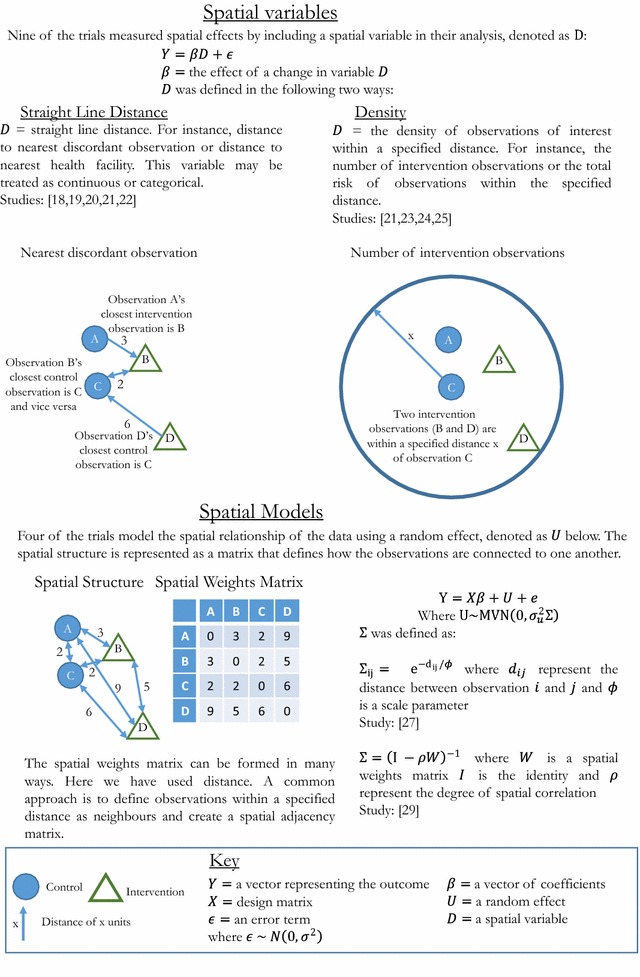
Spatial analysis methods

The studies took two approaches for analysing spatial effects, referred to in this paper as spatial variables and spatial models, expanded upon in Fig. 2. Nine of the trials analysed the effect of a spatial variable which is a measurement that relates to where the observations are located. The two types of spatial variables found in this review were straight line distance such as distance between participants, and density, for instance the number of treated participants within a 100 m radius. Four trials used spatial models by including the spatial structure of the participants using random effects statistical models. The spatial models are specified by measuring how participants are connected to one another, for example recording participants who are neighbours, described further in Fig. 2. Incorporating spatial structure into a model this way treats a spatial effect as an underlying unobserved process which may not be directly measurable. The two approaches make different assumptions about the type of spatial relationship and a variety of methods were used for each approach.

### Spatial variables

#### Straight line distance

Five trials [18–22] estimate spatial effects by measuring the distance between participants and a location of interest. In these studies, the location is either another participant or a feature which may affect the outcome, such as a health facility. Several studies analysed the effect of distance to more than one type of location.

The distance between each control participant to their nearest intervention participant was analysed in three trials [18, 19, 22], termed distance to nearest discordant observation. They also analysed proximity to nearest reservoir or health facility. Distances were categorised and the effect measured for each category. For example, Binka et al. [22] calculated a standardised mortality rate at five separate distance categories.

Kroeger et al. [20] considered whether distance to a participant with the outcome at the beginning of the study affects the odds of having the outcome at the end of the study. They tested at four separate distances and corrected for multiple testing. Ali et al. [21] included distance to school and nearest hospital in a model which assessed the intervention effect and accounted for cluster effects. This was the only trial that included straight-line distance in the primary analysis of their trial.

#### Density

Four trials [21, 23–25] analysed the effect of density in the area surrounding the participants. They analysed the density of factors that may affect the outcome, for example the number of people vaccinated within 100 m. The methods differ by whether they used a count or a proportion and whether they focused on the treatment density or the risk of infection from surrounding individuals. Including density as a spatial variable assumes that number of objects within a certain distance is important as well as the distance to the nearest object.

Lenhart et al. [24] measured intervention density as the number of households with bed nets within 100 m of an observation. The study assessed spatial spillover through the correlation of change in baseline of outcome measure with number of bed net households within 100 m. In contrast, Miguel and Kremer [23] measured density as the proportion of children treated within 6 km of a school as well as the total number and accounted for this in their primary analysis. Ali et al. [21] included a proportion and count density in their primary analysis model by accounting for typhoid prevalence and number of private practitioners for the neighbourhoods of each participant.

Chao et al. [25] differs from the previous applied papers because they develop and test a new method to deal with spatial effects in CRTs. They define a variable called ‘potential exposure’ which is ‘the sum of the relative risks of all who live within 100 m of each person.’ [25]. The ‘potential exposure’ controls for the spatial variation in risk surrounding an observation. They demonstrate that this variable can be used to account for spatially heterogeneous risk factors in the primary analysis of a trial.

### Spatial models

The methods presented in the previous section assume that the underlying spatial process can be measured and spatial effects can be estimated from this measure. The remaining two papers include four trials that model spatial effects using a spatially structured random effect. This approach makes fewer assumptions about the mechanism of spatial process and allows for a range of local and global dependency structures [26].

Alexander et al. [27] investigated the spatial pattern of mosquito borne vectors. Adapted from a previous paper [28] they incorporated a distance parameter in the covariance matrix of a random effect within a negative binomial model [28]. This distance parameter allows participants who are closer together to be more similar than participants that are further apart as shown in Fig. 2. They also estimate a distance decaying parameter in the covariance structure of the random effect, which they use to estimate the “half distance” which is the distance at which spatial correlation halves.

Silcocks and Kendrick [29] applied several types of spatial models to two primary care trials and one simulated trial. The model was a variation of a Besag, York and Mollie model [30] which contains a spatially structured random effect and a random effect for cluster. The spatial structure of the participants was represented using a spatial weights matrix and is included in the covariance of the random effect further described in Fig. 2. In primary care or community based trials people may reside in one area and receive treatment in another [29]. Having a spatial and non-spatial random effect allowed participants to have membership to multiple clusters which they called a multiple membership model. They also consider a fixed north/south and east/west gradient covariate in their model evaluations.

### Impact of spatial effects

All thirteen trials found evidence of a spatial effect within their studies. Seven trials report a protective spillover effect for participants who live close to an intervention. There is evidence that adjusting for spatial effects affects the precision and value of the estimated intervention effect. Chao et al. [25] saw that adjustment for spatial effects lowered the effect estimate of the intervention. The precision and intervention estimate changed in the three trials analysed by Silcocks and Kendrick [29]. The study demonstrated that spatial models fitted better than a standard CRT random effect model by comparing the Akaike information criteria of the models. They are explicit that this is just illustrative but both studies conclude that spatial effects may need to be adjusted for in CRTs and that further research into methods is required. Despite this only two of the nine applied trials adjusted for spatial effects in the primary analysis of their trial.

## Discussion

This review has found multiple approaches to incorporating or measuring spatial effects in the context of CRTs, however these stem from only a few examples in the literature. Further, no conventional or standard approach was found. The approaches differ by whether they directly analyse a spatial variable or model the spatial structure. Spatial variables were either straight-line distance from a participant to a place of interest or a measure of the density surrounding a participant. For instance, distance between a control participant and an intervention participant. Spatial models included spatial structure in the covariance of the random effects model using a distance parameter or spatial weights matrix. Accounting for spatial structure affects both the precision and point estimates of treatment effects and failure to do so could give inaccurate results [25, 29].

The papers in this review are only a small proportion of the total number of CRTs that have been published. That only ten records were found suggests that spatial effects are not often considered in this area. Furthermore, despite evidence of spatial effects, they were rarely adjusted for in the primary analysis of the trial. It appears that the impact of location on analytical approach is at best an afterthought and in most cases ignored. The trials come from a variety of domains and although predominantly focused in infectious diseases, there may be implications for a broader range of trials particularly in trials of health services organisation [29] which are becoming more common [31]. Therefore, a wide range of trials may not be accounting for spatial effects that bias results, however it is presently unclear as to what extent this may be an issue.

Further research is required to determine how much spatial effects impact trial results. Simulation studies may allow exploration of how the magnitude and extent of spatial autocorrelation may bias trial results. There have been some attempts to quantify how important spatial effects may be in trials more generally [32, 33]. Methods that allow for estimation of treatment effects whilst accounting for spatial effects could be investigated and also testing under simulation. However, there are several challenges to overcome such as whether we can estimate the true randomised intervention effect using a spatial model, and how such an effect estimate should be interpreted.

An alternative use of spatial data is to conduct additional analyses to complement analysis of the main trial. These analyses explore spatial component of the trial, and could improve understanding of the mechanism of the intervention effect. Spatial methods could be applied to previous trial data and a toolbox of standard approaches defined allowing future trials to predefine spatial analyses. This would allow for quantification of the distance over which spatial effects are present for different disease areas. A further area of research is in alternative CRT designs, although they are not the focus of this review this might include double randomisation or pseudo randomised trials where clusters are first randomised and subsequently individuals within clusters are randomised to allow for measurement of spillover effects [34, 35].

Multiple terms in the literature refer to spillover effects and many of them could refer to spatial effects with differing terminology between fields and researchers in the same fields. To add to confusion, these terms can have dual meanings for instance, an indirect effect can be the effect on an individual who does not receive the intervention or the effect of an intervention through a mediating variable. The search strategy attempted to include a broad range of terms for CRTs and spatial effects but at present, there is no established standard for citing the use of spatial data in the analysis of CRTs. Consequently, it is possible that trials have been missed. Although this is a weakness, comparison with a larger more general systematic review on health-related spillover in impact evaluations [13] did not result in the addition of any further trials. They had searched 19 databases and screened more than 34,000 records. Due to this the authors conclude the omission of further studies is likely to be minimal.

This review has focused only on the analysis stage of a CRT and it could be argued that adjusting for spatial effects is not necessary in a well-designed trial as clusters should be well-separated to minimise spillover effects [4]. Trial designs such as the fried egg design [3] which incorporates a buffer around clusters could be used to attempt to eliminate or measure spatial spillover. However, in cases where spatial correlation is present over large distances [14] this may not be possible and could lead to the inability to detect the difference between no effect and everyone having an effect [36]. Additionally, as spatial effects are rarely considered in trials, it may not be until after the design stage that the problem becomes apparent, if at all.

On the other hand, having clusters relatively close together could have advantages, because the measurement of spatial spillover effects is of scientific interest. Knowledge that an intervention provides indirect benefit based on proximity is useful to differentiate between interventions and to plan how to benefit the largest number of people. Furthermore, subjects who live further apart are more likely to be heterogeneous than those living close together due to cultural, geographical, and social differences which could make treatment differences harder to distinguish due to imbalance between clusters.

In conclusion although there have been a few attempts to control and estimate spatial effects within the context of human CRTs our understanding is limited. Although there are commonalities between approaches there is no consensus on how to account for spatial effects with in CRTs and more work needs to be done to evaluate and develop spatial methodology within the context of a range of CRTs.

## Additional file

** Additional file 1** Further characteristics for trials in review.

## Abbreviation

CRT: cluster randomised trial
