# Supplementary material for: Spatial analysis of cluster randomised trials: a systematic review of analysis methods
Source: Emerg Themes Epidemiol. 2017 Sep 21;14:12. doi: 10.1186/s12982-017-0066-2 (PMC5609008; doi:10.1186/s12982-017-0066-2)
Supplement: Supplementary file 1 — Additional file 1 Further characteristics for trials in review. [file 12982_2017_66_MOESM1_ESM.docx]

Table S1: Further Details for Trials in the Review

| Author & Year | **Location** | **Design** | **Number of Clusters** | **Cluster Type** | **Number of Individuals** | **Intervention** | **Outcome** | **Notes** |
| --- | --- | --- | --- | --- | --- | --- | --- | --- |
| Binka 1998 [22] | Ghana | Parralel CRT | 96 | Geographical areas | 134,400 | Permethrin-impregnated bed net | Child mortality |  |
| Alexander 2003 [27] | Papua New Guinea | Parralel CRT | 14 | Village | 2,219 | Diethylcarbamazine (DEC) plus ivermectin versus DEC alone | Spatial distirbution of wuchereria bancrofti and microfilaraie |  |
| Gimnig 2003 [19] | Kenya | Parralel CRT | 19 | Villages | 17,000 | Permethrin-treated bed net | Spatial distribution of malaria vectors |  |
| Hawley 2003 [18] | Kenya | Parralel CRT | 79 | Villages | 55,000 | Permethrin-treated bed net | Child mortality |  |
| Miguel & Kremer 2004 [23] | Kenya | Stepped Wedge | 75 | Schools, (Groups) | 30,000 | Deworming | Helminth infection | 75 Schools were split into 3 groups for each wave of the stepped wedge trial |
| Kroeger 2006 [20] | Mexico and Venezuela | Parralel CRT | 18, 18 | Geographical areas | 4,743 (1,095 houses), 5,306 (1,122 houses) | Curtain and water container covers | Dengue vectors |  |
| Ali 2007 [21] | Vietnam | Parralel CRT | 25 | Communes | 56,076 | Vaccine campaign | Vaccine uptake |  |
| Lenhart 2008 [24] | Haiti | Parralel CRT | 18 | Geographical areas | 1,017 houses | Insecticide-treated bednets | Dengue vectors |  |
| Silcocks 2010 [29] | UK | Parralel CRT | (1) 56 (2) 18 (3) 44 | Geographical areas | (1) NA (2) 1,543 (3) 982 | (1) Sun exposure (2) home safety intervention (3) intervention to reduce baby walker use | (1) Lip Cancer (2) number of injuries per individual (3) ownership of baby walker |  |
| Chao 2015 [25] | India | Parralel CRT | 80 | Geographical areas | 62,756 (11,504 houses) | Typhoid vaccine | Vaccine effectiveness |  |
